# Stable isotopes in water vapor and rainwater over Indian sector of Southern Ocean and estimation of fraction of recycled moisture

**DOI:** 10.1038/s41598-018-25522-5

**Published:** 2018-05-15

**Authors:** P. Rahul, K. Prasanna, Prosenjit Ghosh, N. Anilkumar, Kei Yoshimura

**Affiliations:** 1 Divecha Centre for Climate Change, Indian Institute of Science, Bangalore, India; 2 Centre for Earth Sciences, Indian Institute of Science, Bangalore, India; 3 Birbal Sahni Institute of Palaeosciences, Lucknow, India; 4 National Centre for Antarctic and Ocean Research, Goa, India; 5 Atmosphere and Ocean Research Institute, University of Tokyo, Kashiwa, Japan

## Abstract

Stable Hydrogen and Oxygen isotopic composition of water vapor, rainwater and surface seawater show a distinct trend across the latitude over the Southern Indian Ocean. Our observations on isotopic composition of surface seawater, water vapor and rainwater across a transect covering the tropical Indian Ocean to the regions of the Southern Ocean showed a strong latitudinal dependency; characterized by the zonal process of evaporation and precipitation. The sampling points were spread across diverse zones of SST, wind speed and rainfall regimes. The observed physical parameters such as sea surface temperature, wind speed and relative humidity over the oceanic regions were used in a box model calculation across the latitudes to predict the isotopic composition of water vapor under equilibrium and kinetic conditions, and compared with results from isotope enabled global spectral model. Further, we obtained the average fraction of recycled moisture across the oceanic transect latitudes as 13.4 ± 7.7%. The values of recycled fraction were maximum at the vicinity of the Inter Tropical Convergence Zone (ITCZ), while the minimum values were recorded over the region of subsidence and evaporation, at the Northern and Southern latitudes of the ITCZ. These estimates are consistent with the earlier reported recyling values.

## Introduction

The global moisture and heat transport are controlled to a large extent by the oceans that cover 75% of the earth’s surface layer. The Southern Ocean (SO) in particular plays a dominant role in heat and moisture transport process and thereby regulate the planetary scale climate^1,2^. Water recycling process is an important mechanism that moisten the lower tropospheric regions of the atmosphere^3^. The contribution of recycled moisture to the atmosphere especially over the ocean, is poorly constrained owing to lack of observations^3–6^. Moisture on top of the ocean is generated by the process of evaporation which is designated as the first component of water in a hydrological cycle. The processes of evaporation, precipitation and recycling over the oceanic regions can be well understood measuring the stable isotope ratios in both the rain water and water vapor (^18^O/^16^O and D/H)^7–9^. The compostion of initial water vapor fromed from evaporation over the ocean is subsequently modified by mechanisms like rainout, advective mixing and rainfall recycling. The isotope ratios (^18^O/^16^O, D/H) in water is expressed in delta notation (*δ*) where

1 $$\delta ({}^{18}O\,or\,D)=(\frac{{R}_{SAM}}{{R}_{STD}}-1)$$

${R}_{SAM}={}^{18}O/{}^{16}O$ or D/H in samples and *R*_*STD*_ is the same for standard which is the Vienna Standard Mean Ocean Water (VSMOW) provided by International Atomic Energy Agency (IAEA). The d values are multiplied with 1000 and expressed in permil (‰). The δ^18^O and δD of water vapor is dependent on the temperature during phase transformation and can be estimated at equilibrium condition following the equations of isotopic fractionation^10–12^. The process of rainout over ocean that modifies the original vapor isotopic composition and can be predicted using the Rayleigh distillation model^13^. This process involves independent estimation of the cumulative rainout fraction from the initial cloud mass. While δ(^18^O/D) in the vapor over the ocean is solely governed by the temperature and wind speed, while the relative humidity over the evaporating surface drives the d-excess (d-excess, d = δD-8 × δ^18^O)^14^ value^9,15^. The vapor isotopic composition on equilibirum condition depends upon temperature regulated evaporation of surface ocean water and the wind induced kinetic effect is dervied by Merlivat and Jouzel^11^; hereinafter referred to as MJ79 (details in the Methods section). Jouzel and Koster^16^ had pointed out that the closure assumption of MJ79 creates a systematic bias especially in case of local scale application and recommended use of Global Climate model “fitted with isotope tracer diagnostic” for accurate predictions. Such models^17–20^ can represent the process of evaporation better by taking into account the dynamical processes such as moisture recycling and advection besides evaporation alone.

A reasonable agreement exists among the observed relationships between the relative humidity (*rh*) at the moisture source and d-excess as documented in the water vapor isotopic studies over oceanic regions across the globe^9,15,21–25^. Several of the above mentioned observations are either from a single station or spatially spread across small latitudinal transects with exclusive coverage over the Southern Ocean. Uemura *et al*.^9^ and Liu *et al*.^25^ addressed the latitudinal distribution of water vapor isotopic ratios over the Southern Ocean. The role of kinetic fractionation during evaporation and moisture recycling was included as driving parameters in the defining the isotopic ratios of water vapor in their studies.

In the present study 14 water vapor, 15 rainwater, and 35 surface seawater samples were collected during the SO expedition for the year 2013 (Supplementary Table 1**)** and were analyzed for δ^18^O and δD. As a first segment of this study simultaneous observation of SST, wind speed and humidity were compared with the d-excess to draw relationship with physical factors. Further this was extended to the observation with revised estimates accounting for ‘kinetic effect’ using MJ79 formulations and an isotope enabled general circulation model (IsoGSM). Secondly, by utilizing the MJ79 and Rayleigh distillation processes we estimated the fraction of recycled moisture fraction over the tropical Indian Ocean and adjacent SO.

## Background information of the study region

**Figure 1:**
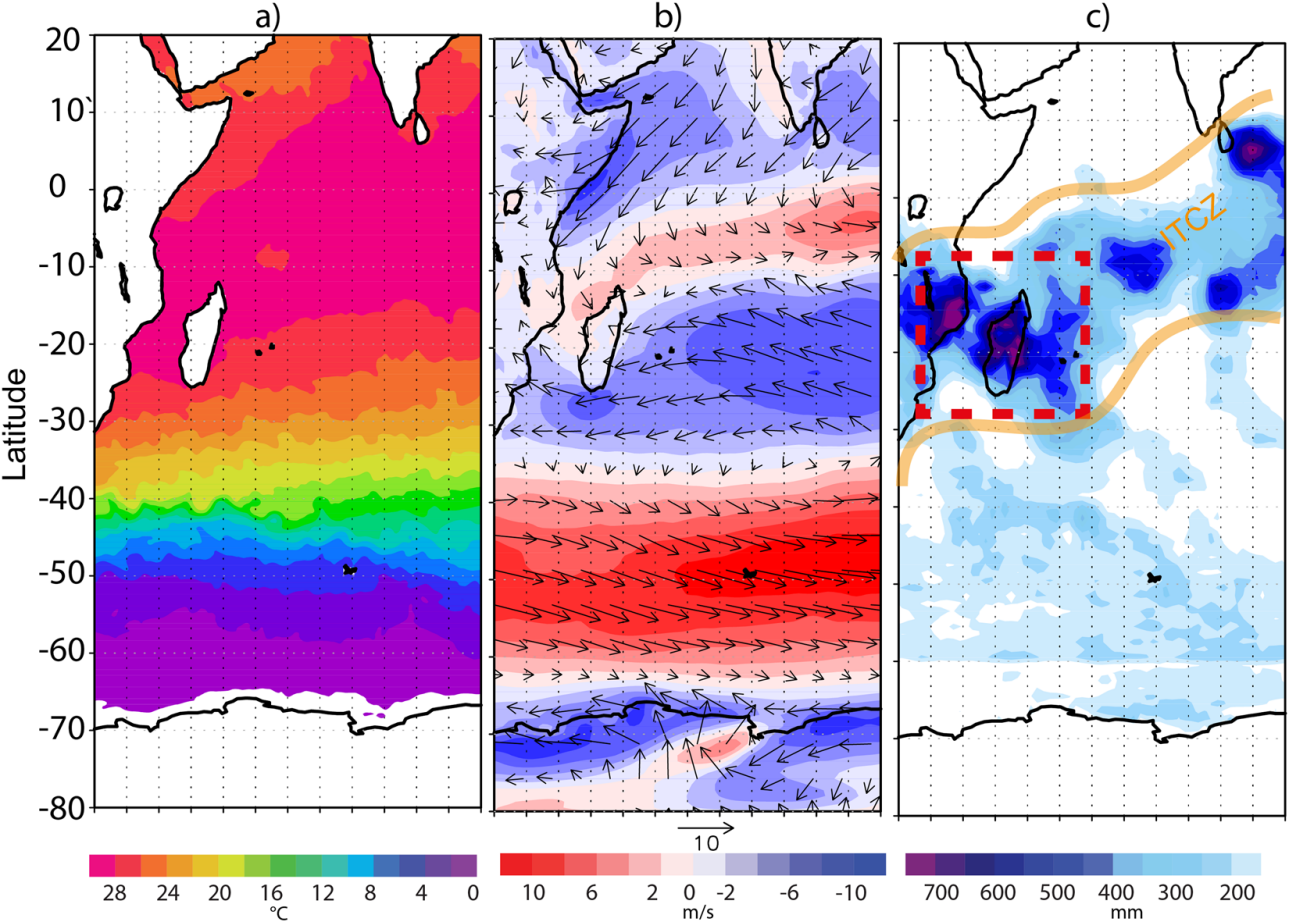
The mean value of SST (**a**), zonal wind speed and wind vectors (**b**) and cumulative rainfall (**c**) over the Indian ocean and the Southern ocean for the months of January - February of the year 2013 are shown here. The location of ITCZ band over the Indian Ocean is marked [in yellow] along with the region of high convective rainfall [in red dashed box]. (The figure is generated using open source free software GRaDS available at http://cola.gmu.edu/grads/downloads.php. The map and International boundaries are only indicative and as provided by the software).

Our study region spans from the tropics (2°S) to the Southern sector (56°S) of the Indian Ocean (IO). The sampling was undertaken during the period 20 January – 28 February for the year 2013 onboard *ORV Sagar Nidhi* (NIOT, Government of India) at stations, more or less equally spaced, along with the sailing transect (Supplementary information 1). The spatial mean states of SST, wind (magnitude and direction) and rainfall during the period of sampling are shown in Fig. 1. From 0°S to 30°S the SST (Fig. 1a) varied from maxima of 30 to 26 °C. The zonal wind direction (u) shows the dominance of westerlies wind vectors over this region suggesting the origin of moisture from the regions of Northern Indian Ocean (Arabian Sea and Bay of Bengal) (Fig. 1b**)**. Further, at around 20°S, the dominance of easterlies with the wind velocity reaching the value of ~10 m/s was recorded. A backward HYSPLIT model S4 trajectory analysis at sampling locations is provided in the Supplementary information 3, which agrees with the shipboard observations shown in Fig. 1.

A region of strong convection can be observed in the zone of convergence of wind vectors, which lies between 10°S to 20°S. The cumulative rainfall plot (Fig. 1c) shows the occurrence of intense rainfall activity on or near the ITCZ covering latitudinal extent between 10°S and 20°S. The enclosed red dashed box shows Madagascar region that experiences high rainfall activity due to buildup of a low pressure system and orographic effect during the months January-February^26^. A large drop in the average SST value from 25 °C to 0 °C was recorded between 30°S and along the progressive journey up to 60°S latitude. The region lying between 40°S to 60°S marks the region of westerlies with highest wind speed in the entire SO, popularly known as the ‘roaring forties’. The transition zone is defined by a region lying between 30 ° to 40°S where the orientation of trade wind vectors changes from easterlies to westerlies.

The surface seawater based earlier isotopic studies across the latitudes from the tropics to Antarctic coast over this sector showed a distinct zonation across SO, where the role of physical factors like evaporation, precipitation and melting/freezing varies^27,28^. Srivastava *et al*.^27^ has demarcated the region north of ~4.5°S as the zone of evaporation, while the region lying between 4.5 to 20°S is marked as the zone of precipitation (under the influence of ITCZ). Evaporation dominating region lies between 20 to 41°S zone. The region lying between 41°S to 47°S is known as the transition zone, sandwiched between the zone of evaporation and the melting or freezing and confined between 47°S to 68°S. Recent study by Tiwari *et al*.^28^ using Salinity- δ^18^O relationships over the Southern Ocean regions have also recorded similar character of surface water across different frontal systems for other years.

## Results and Discussions

### Zonal changes in the physical parameters and isotopic composition across latitudes

**Figure 2:**
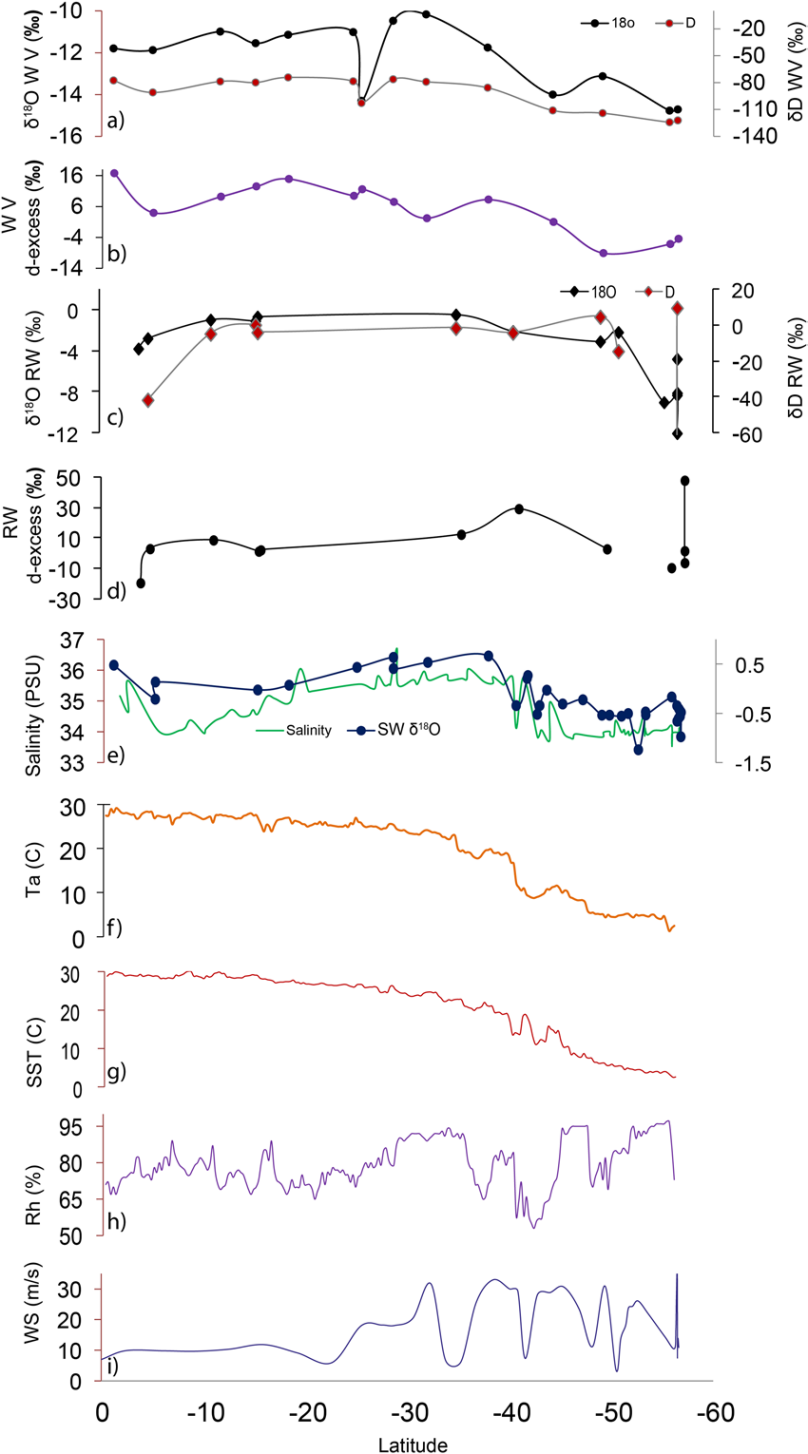
The latitudinal variability isotopic composition along with measured physical parameters of ocean and atmosphere are shown in the plot. The δ^18^O and δD of water vapor (**a**), water vapor d-excess (**b**) δ^18^O and δD of rainwater (**c**), rainwater d-excess (**d**) and seawater (**e**) along with other physical parameters such as Salinity (**e**), Air temperature [Ta] (**f**), Sea Surface Temperature[SST] (**g**), Relative humidity (Rh) (**h**) and Wind speed (WS), (**i**) observed during the sampling period along the cruise track.

In this section, we have described the observations of isotopic variability, salinity, SST, wind speed, relative humidity, air temperature recorded during the period of our study. The δ^18^O and δD isotopic values together with onboard observations of air temperature, sea surface temperature, relative humidity and wind speed are plotted for comparison (as shown in Fig. 2). The details about the sampling procedure and analytical methods are provided in the Supplementary information 1. The range and average isotope ratio of vapor, rainwater and seawater are mentioned in Table 1. The water vapor δ^18^O values varied from −14.8‰ to −10.2‰ with an average value of −12.3 ± 1.6‰, whereas δD values varied from −124.3‰ to −74.2‰ with an average value of −92.6 ± 18.4‰. For rainwater, the δ^18^O values varied from −12.1‰ to 0.5‰ with an average value of −4.8 ± 4‰, and the similarly δD values ranging from −103‰ to 9.4‰ with an average value of −24.9 ± 36.9‰. The d-excess value observed for the water vapor samples showed a range from −9.1 to 14.9‰, where the average was 5.5 ± 8‰, and the average d-excess value for rainwater that showed a range between −19.2 to 47.8‰ was 6.3 ± 17.6‰. The d-excess estimated for the water vapor were lower than the equilibrium value of 10‰, with only a few exceptions where higher values (16.7, 12.5, 14.9 and 11.6‰) were observed. Rainwater d-excess mostly showed a trend of lowering of values across the latitudes, indicating rainfall evaporation and recycling^8,29^, whereas only very few samples showed higher d-excess values (12.5, 29.3 and 47.8‰). An identical latitudinal trend is observed for the variations in sea surface salinity and sea surface water isotopic composition (Fig. 2e). The ITCZ marks a region dominated by precipitation, where salinity was low (34 PSU) for the surface ocean water. This was also seen in the lower δ^18^O values measured in the rainwater (−3.8 to −1.1‰) (Fig. 2c) that resembles precipitation isotope values in the tropical convective rains^30^. The vapor δ^18^O values in this region lie between −11.8 to −10.9‰. Interestingly, a sample of vapor collected from 35°S showed an anomalously low δ^18^O value of −14.3‰ and high d-excess values, both indicating involvement of recycled moisture^7,29,31^. The higher d-excess values in vapor can be explained by the kinetic evaporation, under humidity deficit condition, that tends to increase the d-excess of the vapor and lower the δ^18^O^9,32^. It can be noted that the water vapor samples with high d-excess values (11.6, 12.5, 14.9 and 16.7‰) mostly lie in the region of ITCZ indicating the role of active recycling process.

**Table 1:** The maximum, minimum and average value of the isotopic compositions of water vapor, rainwater and surface water samples collected during the expedition.

	Water vapor			Rainwater			Surface water		
	Min	Max	Avg (±St.Dev)	Min	Max	Avg (±St.Dev)	Min	Max	Avg (±St.Dev)
δ^18^O	−14.8	−10.2	−12.3 ± 1.6	0.5	−12.1	−4.8 ± 4	−1.24	0.66	−0.22 ± 0.46
δD	−124.3	−74.2	−92.6 ± 18.4	9.4	−102.9	−24.9 ± 36.9	—	—	—
d-excess	−9.11	16.7	5.53 ± 8	−19.2	47.8	6.3 ± 17.6	—	—	—

The water vapor shows enriched δ^18^O values (−10.2‰) southern side of 30 °S latitude coinciding with the region of evaporation^27^ following the enrichment in seawater δ^18^O (0.66‰). The salinity and δ^18^O values decreased beyond 35°S which is marked as the transition zone, where processes like melting/freezing are active^27,28^. The lowest δ^18^O values for seawater (−1.2‰) and rainwater (−12.1‰) were recorded in the sub-polar region, beyond 50°S latitude.

The variability of physical factors in the overlying atmosphere and surface seawater was smaller for the region between equator and 30°S during the summer of 2013, representing a region with a near homogeneous behaviour (Fig. 2f,g). For example, the SST varied from 29.9 to 23.6 °C between 1 to 30°S latitudes. This was consistent with the air temperature variability that showed a range of 29.2 to 23.5 °C over this region. A monotonic drop in SST and air temperature was recorded south of 30°S latitude. Observation suggests drop in SST value from 24.7 to 2.5 °C consistent with the drop recorded in the air temperature *i*.*e*., 24.2 to 1.3 °C. A large shift in the relative humidity (*rh*) and wind speed values (Fig. 2h,i) were also recorded over the southern latitudes especially in the subtropical and sub-polar regions. The effect of oceanic conditions on the isotopic composition of water vapor is a matter of detail investigation that has been discussed in later sections.

### Influence of sea surface parameters on water vapor δ^18^O

**Figure 3:**
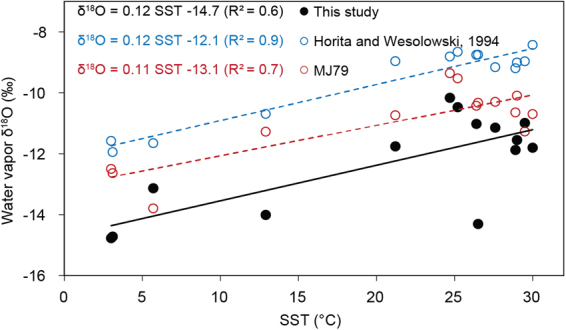
The plot shows comparison between observed water vapor δ^18^O [black line] with the same calculated using equilibrium fractionation [Horita and Wesolowski, 1994; blue dashed line] and MJ79 formulation [red dashed line].

The sea surface temperature and relative humidity conditions control the fractionation of isotoes in the water vapor as it transform from water during evaporation^9,13,15^. The role of temperature in determining δ^18^O variability observed in the water vapor samples from the tropical to polar regions can be explained using the fractionation equation^33^. The δ^18^O values of vapor showed a significant correlation with SST (R^2^ = 0.6, p < 0.025). (Fig. 3) SST explains 60% of the variability recorded in the vapor δ^18^O while the role of other physical parameters on vapor δ^18^O remained unresolved. We evaluated the water vapor δ^18^O invoking both equilibrium and non-equilibrium fractionation effect and compared with the observed δ^18^O values. Our calculation involves two assumptions: (1) At equilibrium condition surface seawater temperature determines the isotopic composition of resultant vapor as given by Horita and Wesolowski^33^ in the following equation;

2 $${10}^{3}\,{\rm{In}}\,{\rm{\alpha }}\,({}^{18}{\rm{O}})=-\,7.685+6.7123({10}^{3}/{\rm{T}})-1.6664({10}^{6}/{{\rm{T}}}^{2})+0.35041({10}^{9}/{{\rm{T}}}^{3})$$

and (2) by the MJ79 model (Methods, equation 6) where the observed SST, seawater δ^18^O, wind speed and normalized relative humidity values pertaining to the time of sampling were useful for deducing the δ^18^O of water vapor and rain.

The vapor δ^18^O composition estimated using observed SST values under the two assumptions are plotted in Fig. 3. The slopes of the regression lines are similar; however, the intercept values of the regression equations for the calculated δ^18^O composition are different due to the difference in the involvement of kinetic process. The equilibrium model estimates showed enriched intercept value (−12.1‰) whereas the MJ79 (with kinetic fractionation using wind speed over the ocean being incorporated) showed a lower intercept value (−13.1‰). The depletion of the MJ79 values suggests the role of wind driven kinetic fractionation, which is more pronounced in the water vapor observations where an intercept of −14.7 is obtained with SST. The effect of kinetic factors can be further investigated using d-excess values which are discussed in the section later.

### d-excess relationship with relative humidity and SST

**Figure 4:**
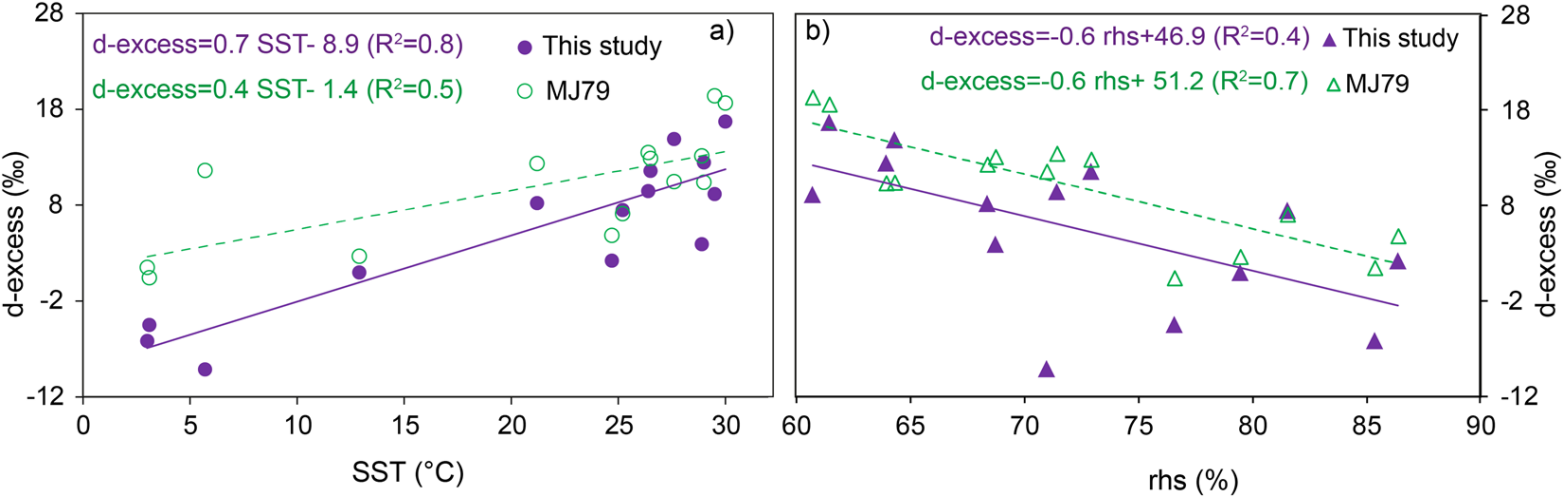
The relationship between d-excess and SST (**a**), relative humidity normalized to surface temperature [*rhs*] (**b**). In the plots violet color markers are the observed samples and green markers are values obtained from MJ79 formulation.

Kinetic process governs the relationship between d-excess with SST and rhover evaporation ocean surface^13^. The MJ79 formulation suggests that d-excess value is positively correlated with SST with a gradient of 0.35‰/°C whereas it is inversely correlated with relative humidity with a gradient −0.45‰/%^9,34^. The SST influence on d-excess is via involvement of equilibrium and kinetic fractionation during phase transformation. Humidity effect is caused due to the gradient of moisture between the micro layer over the water surface and that of air on top. The process is driven by the molecular diffusion from the surface to the overlying atmosphere at the air-sea interface, which is governed by wind strength and temperature as described in Craig-Gordon model^10^. Lower d-excess value in water vapor on top of the ocean is recorded in cases of rough ocean conditions^9,15,25^ that generates sea spray due to wave breaking. The effect was pronounced near polar and subtropical regions where frequent extra-tropical cyclones were recorded during the cruise time. Previous studies over the regions of SO^9,25^ have shown a significant dependence of d-excess with SST and normalized relative humidity (*rhs*) (see Methods). In the present study we observed a significant correlation coefficient (R^2^) while comparing the water vapor d-excess and SST values (Fig. 4a) (R^2^ = 0.8, p < 0.01) and the best fit equation is given by d-excess = 0.7 SST − 8.9. Similarly, d-excess values also showed dependency with *rhs* (R^2^ = 0.4, p < 0.025) and the best fit equation is given by: d-excess = −0.6 *rhs* + 46.9 (Fig. 4b).

The water vapor d-excess values were estimated using MJ79 model using the observed physical factors (wind speed and *rhs*). The estimated d-excess values fitted against the observed SST using an equation: d-excess = 0.4 SST – 1.4, (R^2^ = 0.5) (Fig. 4a**)**. Similarly, the d-excess values plotted against *rhs* gives the equation: d-excess = 0.6 *rhs* – 51.2(R^2^ = 0.7, p < 0.01) (Fig. 4b). The linear regression lines defining the model and observed values tend to merge near the tropical belt where higher SSTs were recorded. The region of tropics is characterized by higher SST and low temporal variability (confining to the period of sampling) of SST and other physical factors. However, a large difference in d-excess values was recorded over the region of colder SST (<20 °C). The role of cyclonic activity and presence of sea spray were factors attributed for the samples recording low d-excess values^9^. In case of *rhs* and d-excess relationship, the two regression lines deduced from the models and from observation follow a parallel trend with a small offset in the intercept values. The difference in the intercept values is due to lack of consideration the processes like rainout and recycling in the MJ79 based d-excess estimation. As suggested by Jouzel and Koster^16^, the MJ79 parameterization leads to bias due to its closure assumption. This is documented in Fig. 4a,b as the differences in the intercepts. The d-excess-*rhs* relationship based on our observations is in agreement with other observation by earlier workers^9,34^. However, in the present study, the variance (60%) is mostly explained by SST rather than by *rhs* (40%). As discussed previously, the MJ79 calculation is *in-situ* and can have a reasonable bias which can be minimized in a dynamical model like IsoGSM.

### Comparison with IsoGSM data

**Figure 5:**
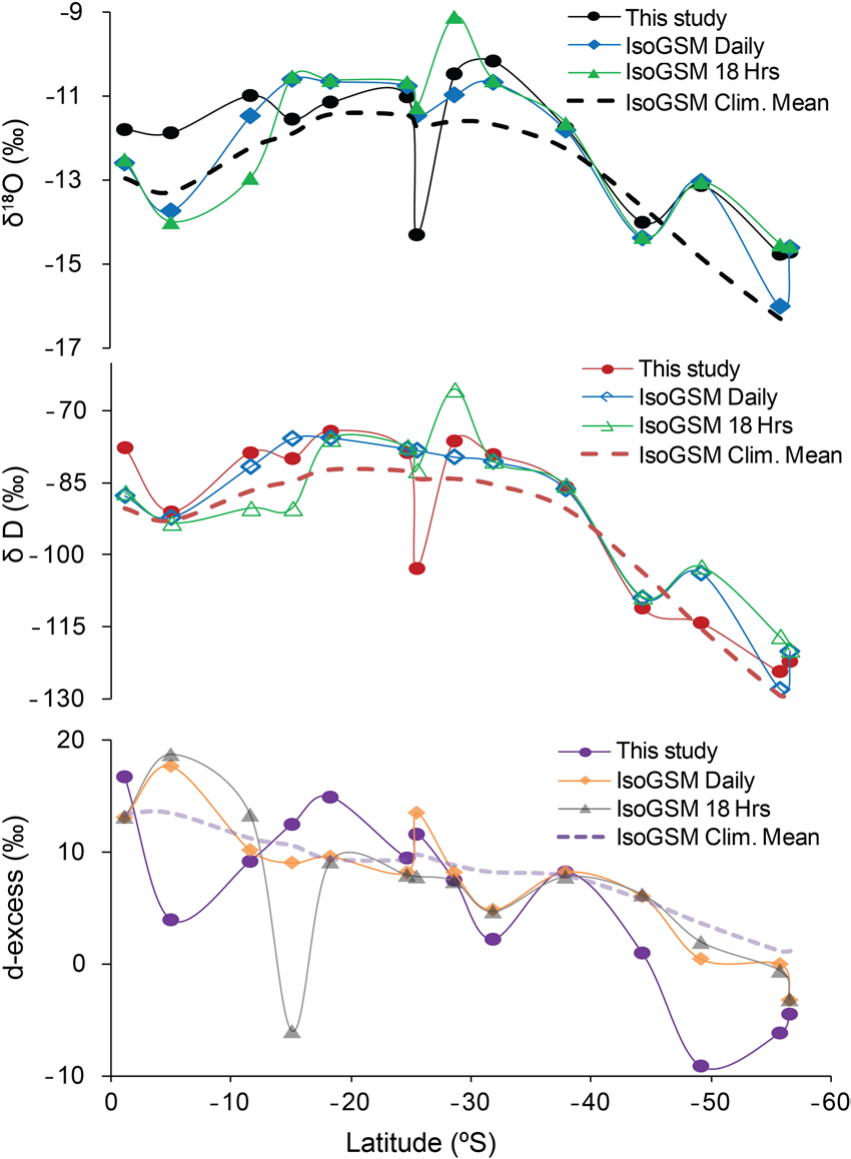
The plot shows the latitudinal variations of observed water vapor δ^18^O, D and d-excess data compared with that of IsoGSM model data. The IsoGSM data are obtained for three different time scales: daily mean, 6 hourly and the climatological mean obtained for 34 years [from 1979 to 2013]. The time corresponding to 18:00 hours in the IsoGSM 6 hourly dataset are used here as it is the closest to our sampling time of water vapor.

**Figure 6:**
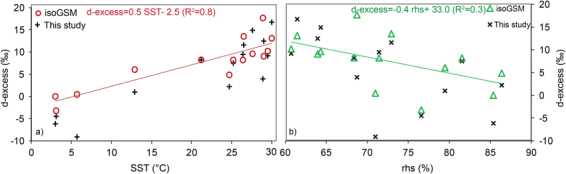
The IsoGSM d-excess relationship with SST and *rhs* are shown here in (**a**) and (**b**) respectively. In the plots the observation points are shown as black markers. It can be noted that the observed samples and IsoGSM data shows good agreement indicating the model predictability of water vapor isotopic composition over oceanic region.

Isotope enabled global spectral model (IsoGSM)^19^ incorporates both kinetic and equilibrium fractionation during determination of isotopic ratios in the vapor after taking into account the complex atmospheric processes via spectral nudging technique. Here we compare our observations with the IsoGSM based predictions at three time scales; 6 hourly data, daily mean and climatological mean (1979 to 2013). The climatological mean gives the broad representation of the latitudinal variability during the multi-decadal period of 34 years. The six hourly data is generated in the IsoGSM model corresponding to the time 0, 6, 12 and 18 hours. Since our sampling was from 14:00 hours to 17:00 hours we selected the IsoGSM data of 18 hours integration for the best comparison. The latitudinal comparison of δ^18^O, δD and d-excess between observed values and IsoGSM model predicted values show an excellent match (Fig. 5**)**. Daily comparison of observed δ^18^O, δD and d-excess with IsoGSM showed R^2^ values of 0.6,0.8 and 0.61 respectively and the same for hourly data showed R^2^ values of 0.5, 0.8 and 0.2. However, the d-excess values showed inconsistency, indicative of poor representation of the recycled moisture component in the IsoGSM model. The latitudinal variance observed for the daily as well as hourly data of IsoGSM model showed a similar feature as that of the climatological mean. The mean of the absolute difference between the model daily data and corresponding observed data for δ^18^O, δD and d-excess values are 0.75, 0.48 and 3.9‰ respectively, whereas for hourly model data the differences are 0.89, 6.6 and 5.5‰ respectively. The largest difference was noted for the anomalous sample where a drop of 3.3‰ in observed δ^18^O value compared to the decrease in the IsoGSM model of 1.3‰. The deviation in the d-excess from the model predicted value was found the maximum for the region lying in the Polar latitudes. The highly depleted d-excess value clearly stands out as an outlier point in both the plots (Fig. 6a and b). The d-excess-SST and d-excess-*rhs* linear relationship based on daily data of IsoGSM model and the observed water vapor values (that are plotted in Fig. 6**)** shows agreement with the observations (0.5 SST − 2.5, −0.4 *rhs* + 33).

### MJ79- Rayleigh processes based model for latitudinal variability of rainwater and water vapor isotopic composition

**Figure 7:**
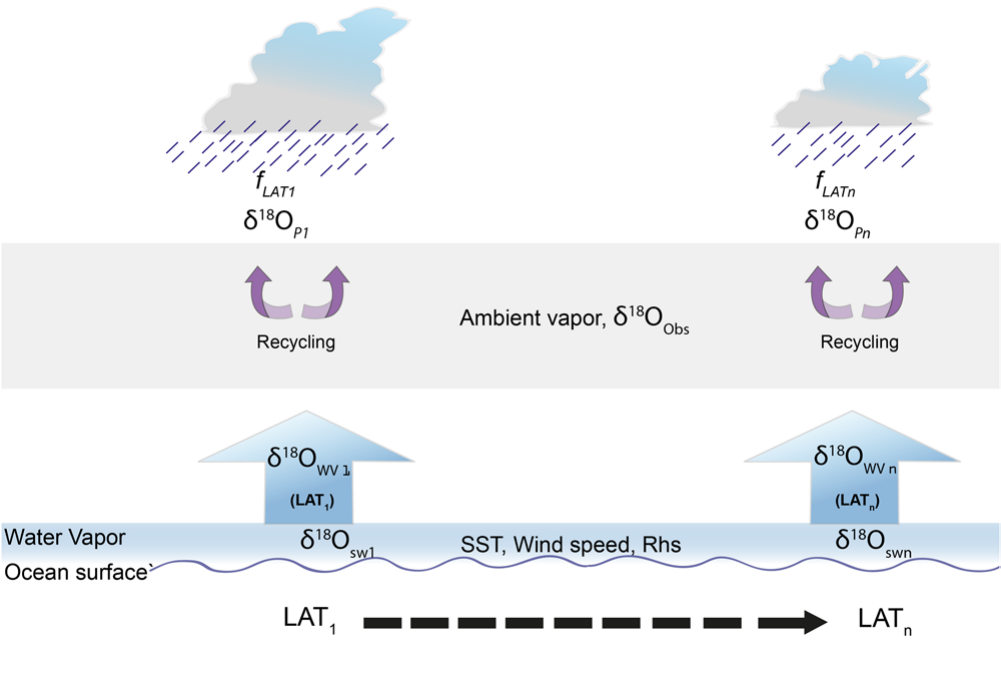
The figure shows the process used in the model calculation under section 2.7. Observed values of SST, wind speed,* rhs*, δ^18^O in sea water [δ^18^O_sw_] over different latitudes are used as the initial condition for the water vapor isotopic composition of surface evaporative flux [δ^18^O_wv_]. The rainout fraction [*f* ] is used to find the rainwater isotopic composition and the remnant water vapor. The ambient vapor is the vapor collected during sampling [δ^18^O_Obs_] which includes the evaporated, transported and recycled components.

In this section, we have used the MJ79 formulation combined with rainout process using the Rayleigh equation for predicting the isotopic composition of water vapor and rainwater from the tropics to the polar region. Figure 7 shows a schematic process in the model calculations. The primary evaporated water vapor isotopic composition was obtained as described in Methods section where observed SST, wind speed, *rhs* and isotopic values of seawater were used as input parameters. This water vapor is assumed to be well mixed to form the cloud and subsequent rainout by condensation. The rainout fraction (*f* ) over each location is estimated based on satellite and reanalysis data set (Supplementary information 2) which is defined as,

3 $$f=RF/PWC$$

RF is the rainfall amount and PWC is the total precipitable water content of the overhead atmosphere. The *f* was calculated in a 2.5 × 2.5 degree box keeping the sampling station at the center. The temperature of condensation was derived by taking into consideration the cloud height observed near the tropical and over high latitude region. The average cloud height over the tropical Indian Ocean is ~8 km, while the average cloud height reduced to ~3 km beyond 40°S latitudes as evident from the ENVISAT global cloud top height data^35^. A large shift in cloud heights is expected to change the temperature of condensation. To estimate the condensation temperature we have chosen mid level *i.e.* middle point of the lower surface and the cloud top height and evaluated mean temperatures over the tropics and sub-tropical regions. For this approach, we selected 700 mb pressure level over the tropical regions (0–35°S) as elevation for condensation of vapor with estimated average temperature value of 8 ± 1.7 °C whereas for the regions beyond 40°S latitudes 850 mb was chosen with an average temperature value of −2.7 ± 3 °C. These pressure levels correspond to an altitude of 3000 m and 1500 m above mean sea level in the tropics and polar region respectively. The condensation temperatures and water vapor isotopic ratios were used as input parameters in the Rayleigh’s fractional distillation model to obtain the rainwater and the remnant vapor isotopic ratios as following;

4 $${R}_{fwv}={R}_{wv}{f}^{(\alpha -1)}$$

where *R*_*fwv*_ is the vapor isotopic ratio after rainout, and *R*_*wv*_ is the initial source vapor composition. Here *f* is the remaining fraction of moisture after rainout (corresponding to each day of sampling), and α is the equilibrium fractionation factor during condensation (α > 1).

A comparison of rainwater isotopic ratios estimated using the formulation with the observed values showed excellent match (Supplementary information, Figure S4**)**. In the above estimates, we assume that the variability of water vapor isotopic composition remained constant along the zonal band having a spread of 5° latitude. This is further supported by our observations where the temperature and wind speed data in the east-west longitudinal transect were found nearly constant (Fig. 1a,b**)**.

The lighter values recorded in the ambient vapor compared to the same day estimated values suggests a variable contribution of recycled moisture fraction. This fraction in terms of percentage can be calculated from the Rayleigh distillation formulation as following,

5 $$\mathrm{ln}\,{f}_{r}=\frac{\mathrm{ln}\,{R}_{model}-\,\mathrm{ln}\,{R}_{obs}}{(1/\propto -1)}\times 100$$

where *f*_r_ is the recycled fraction; *R*_*ob*s_ and *R*_*model*_ are observed and modeled isotopic composition of water vapor respectively; α represents the fractionation factor corresponding to the air temperature across latitudes which is associated with liquid to vapor phase transformation^33^.

**Figure 8:**
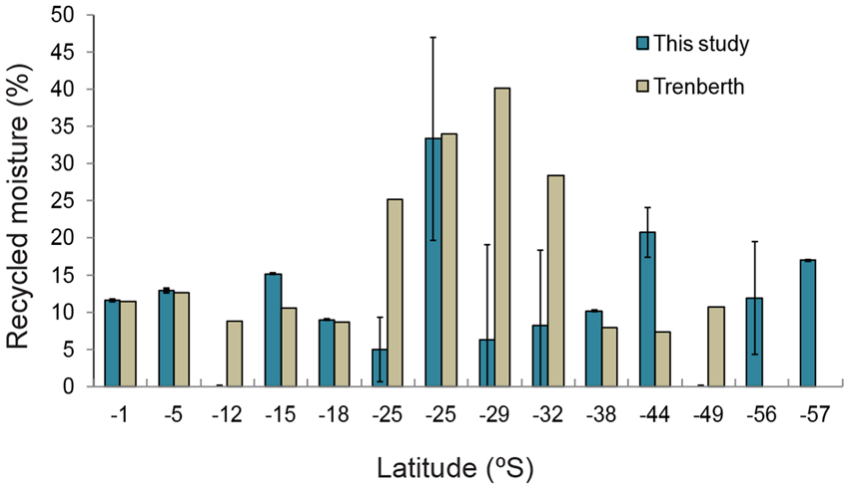
The recycled moisture percentage in our study (horizontal bars) is calculated based on water vapor isotopic composition across latitudes for the austral summer months of 2013. The red markers shows the long term mean annual values of recycled moisture obtained from Trenberth^3^ over the oceanic regions covering our sampling location. The differences in both the studies can be attributed to the difference in the time scales.

Our results suggest variation in *f*_*r*_ from 0 to 33.0% with an average value of 11.5 ± 8.5% (Fig. 8). The maximum value observed lies in the vicinity of the ITCZ (at 25°S) where the observed rainfall was also found the maximum. The higher chances of convergence of moisture (and resulting rainfall) and low advection results in the high recycled moisture values^3^. The low values (<15%) were recorded over the region of subsidence and evaporation which lies on the Northern and Southern latitudinal belt in proximity to the ITCZ. The higher precipitation zone over the Polar Region (57°S) also recorded 16.9% moisture recycling where the role of extra-tropical storm was visible. The main uncertainties in our calculation comes from the estimation of average rainout fraction and condensation temperature. We have estimated at each point a range of values with higher and lower envelope being added quadratically to ascertain the total uncertainty as represented by error bars in Fig. 8. The maximum error estimated was ± 13.7%. In order to defend our estimates of the percentage of recycled moisture, we compared our observations with earlier long term observations (1979 to 1995) based on satellite and reanalysis data available till 50°S latitude^3^. Our estimates are in agreement with the Trenberth^3^ over the region of Southern ocean and tropical latitude. The discrepancies about the values can probably be explained by the smaller time duration and large spatial difference of sampling in the present study (January to February) of 2013. Our study provides latitudinal variability of water vapor isotopes and quantified the fraction of recycling which will be useful for better understanding the hydrological cycle over oceanic atmosphere as well as to interpret isotopic composition of surface water in paleo records.

## Conclusion

In this study, we used stable isotope ratios water vapor, rainwater and seawater samples from the tropical Indian Ocean and the Southern Ocean for understanding its dependency on ocean atmospheric parameters. The sampling stations were spread across diverse zones with varying SST, wind speed and rainfall regimes. The latitudinal variation of seawater isotope values showed features characteristic of evaporation and precipitation zones. The water vapor isotope values were compared with predicted values using simple equilibrium fractionation and MJ79 formulation. We used the d-excess parameter in water vapor to investigate its dependency on SST and relative humidity over the oceanic regions. The relationship of d-excess with SST and humidity obtained from our study is: d-excess = 0.7SST−8.9 (r^2^ = 0.8) and d-excess = −0.6 *rhs* + 46.9 (r^2^ = 4). We compared the water vapor isotope results with isotope enabled Global Spectral Model (IsoGSM) and found that the water vapors over the oceanic regions across latitudinal belts are well represented in the model data. We estimated the fraction of recycled moisture at each location using our observation. The fraction varies from 0 to 33.0% with an average value of 13.4 ± 7.7%. The maximum value observed lies in the region of ITCZ near to 25°S where rainfall was found the maximum. The low values (<15%) were recorded over the region of subsidence and evaporation regions which lies on the Northern and Southern periphery of the ITCZ.

## Methods

### Isotopic composition of vapor over ocean

The effective fractionation factor α from liquid water to water vapor over an open surface is a combination of equilibrium and kinetic fractionation. For estimating water vapor isotopic composition we followed the closure assumption that the vapor isotopic composition at the sea surface is equal to the boundary layer composition [Merlivat and Jouzel^11^ (referred to as MJ79); Benetti *et al*.^15^] and thus the effective fractionation can be expressed as

6 $$\alpha ={\alpha }_{eq}[{\alpha }_{k}+rhs(1-{\alpha }_{k})]$$

The stable isotopic composition of water vapor generated by the process over the surface ocean water can be thus obtained as

7 $${R}_{BL}=\frac{{R}_{SW}}{{\alpha }_{eq}[{\alpha }_{k}+rhs(1-{\alpha }_{k})]}$$

where *α*_*eq*_, *α*_*k*_ and *rhs *are the equilibrium fractionation factor, kinetic fractionation factor and relative humidity respectively whereas, *R*_*BL*_ the isotopic ratios of boundary layer vapor under the closure assumption and R_*SW*_ that of sea surface water. Since the evaporation depends on the gradient of humidity between the atmosphere and the micro boundary layer over the ocean surface, it is appropriate to use relative humidity term which is normalized to the SST. The relative humidity values are normalized to the sea surface temperature using an equation:

8 $$rhs=rh\times {e}_{atmos}/{e}_{SST}$$

where *e*_*atmos*_ and *e*_*sst*_ are the values for the saturation vapor pressures at constant air temperatures and SSTs.

The kinetic fractionation factor depends on the wind speed at the ocean surface as follows:

9 $$\alpha kin=\{\begin{array}{ll}\,1-A & for\,ws < 1\,m/s\\ 1-(B\,\times ws+C) & \,for\,ws\ge 7\,m/s\end{array}\}$$

where constants A, B and C are 0.006, 0.000285 and 0.00082 for δ^18^O and 0.00528, 0.0002508 and 0.0007216 for δD (as given by MJ79). The net fractionation due to kinetic process relates with wind speed and normalized relative humidity values.

## Electronic supplementary material

Supplementary information
