# Supplementary material for: Stable isotopes in water vapor and rainwater over Indian sector of Southern Ocean and estimation of fraction of recycled moisture
Source: Sci Rep. 2018 May 15;8:7552. doi: 10.1038/s41598-018-25522-5 (PMC5954022; doi:10.1038/s41598-018-25522-5)
Supplement: Supplementary file 1 — Supplementary information [file 41598_2018_25522_MOESM1_ESM.docx]

Supplementary Information

**Stable isotopes in water vapor and rainwater over Indian sector of Southern Ocean and estimation of fraction of recycled moisture**

**Rahul P, K. Prasanna, Prosenjit Ghosh, Anilkumar N, Kei Yoshimura**

**1) Sampling and analytical methods**

Water vapor, rainwater and surface seawater samples were collected onboard Oceanographic Research Vessel (ORV) Sagar Nidhi during the Indian Southern Ocean expedition of 2013. The sampling stations of water vapor and rainwater are marked in **Figure S1 (top panel).** A total number of 14 water vapor, 15 rainwater, and 35 surface seawater samples were collected across the transect (**Table S1**).

The vapor collection setup was installed in an enclosure located at the focsle of the ship as shown in **Figure S1 (bottom panel)**. For water vapor sampling a polyethylene tubing was connected to the cold trap (Rahul et al. 2016 ) using an Ultra-Torr^TM^ union (Swagelok^TM^ make). The trap consists of three inter-connected tubes (**Figure S2**) to baffle the air flow thereby enhancing the efficiency of moisture trap, simply due to larger surfaces for condensation of the water vapor. The inlet was placed on the compartment rooftop in the downstream direction to avoid contaminants from the ship exhaust (**Figure S2**). The entry part of the inlet tube was attached to a vertically inverted funnel to avoid any rainwater or any other spilled water. Additionally the funnel was cautiously placed behind a square wooden plank to avoid secondary intakes such as rainwater, or any other source of water splashed into the funnel during the ship’s movement. Atmospheric water vapor was trapped by passing the air through a cold trap using a pump.

**Figure S1: The above plot shows the sampling points of water vapor and rainwater collected during the cruise expedition of 2013. The bottom image shows the picture of ORV Sagar Nidihi , where the dashed red box indicate the enclosure where water vapor samples were collected (*Top figure was generated using licensed ArcGIS 10 software. The map and International boundaries are only indicative and as provided by the software The photograph of the vessel was taken by one of the co authors during the expedition*).**
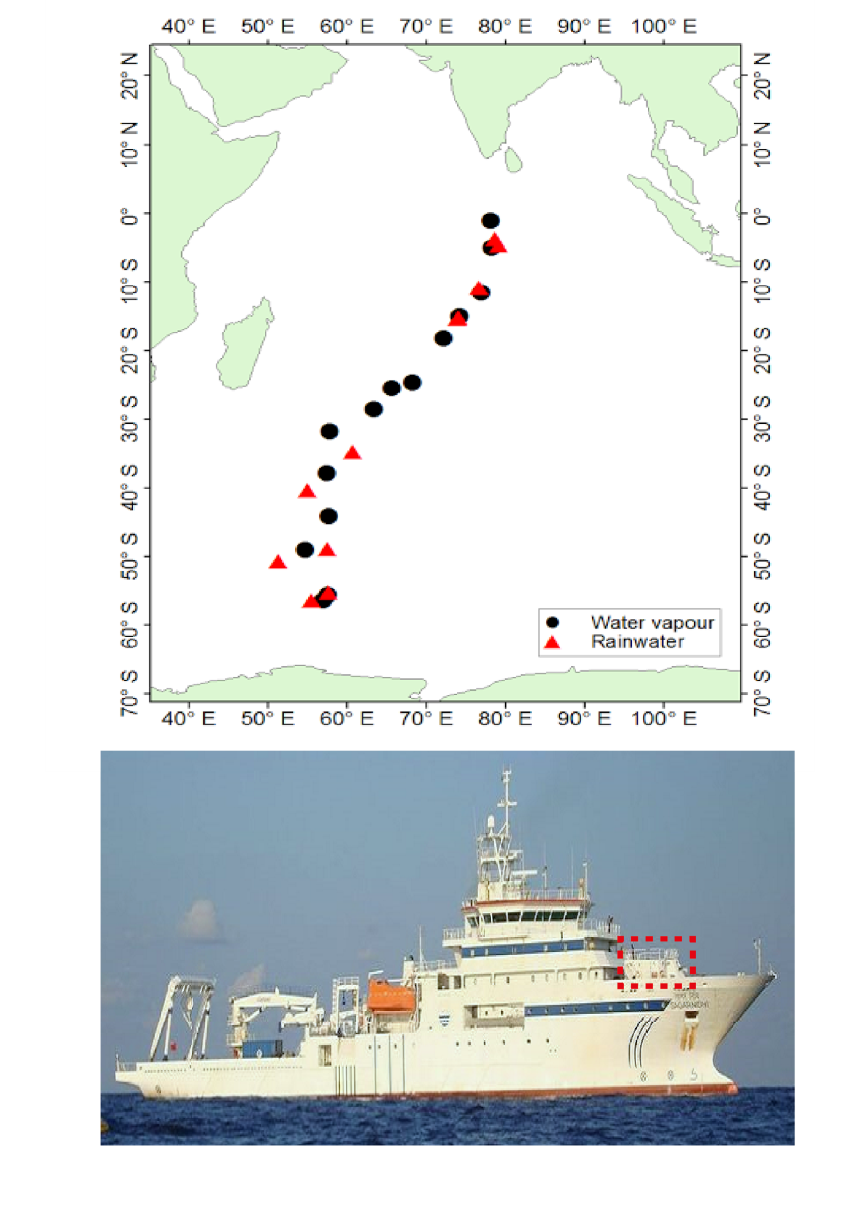


The pump was connected to the outlet of the trap using another Ultra-Torr^TM^ union and air was flushed at a rate of 250 ml/min. The system is initially flushed using dry air before attaching to the trap. Subsequently, the trap was immersed in Ethanol (99.99%) liquid Nitrogen slush at -80 ^o^C and kept for ~20 min to attain stability before starting the pump for sample collection. The sampling time for the vapor collection was fixed at 14:00hrs to 17:00hrs at all the stations, which allowed the collection of well mixed atmospheric boundary layer water vapor.

Rainwater samples were collected using a Teflon^TM^ coated stainless steel funnel attached with a manually operated valve. The outlet of the valve was equipped with an adopter which can be screwed with the sampling bottle; preventing any leakage from the joints. The bottle was disconnected and closed with a cap immediately after the rain to prevent any loss due to evaporation. The salinity was measured using an Auto Salino Meter (Tsurumi Seiki Co. Ltd, Japan) installed on-board. Salinity values are expressed in the 1978 Practical Salinity Scale (PSU) (PSS-78) with a precision of ±0.005 PSU. Sea Surface Temperature (SST) was measured using a bucket thermometer (Theodor Friedrichs and Co, Germany; accuracy ± 0.2°C). Isotope ratios were measured following the equilibration method [*Rangarajan and Ghosh*, 2011] using a Isotope Ratio Mass Spectrometer (IRMS) (Thermo Scientific, MAT 253) coupled with a GasBench II peripheral. The analytical uncertainty of the samples were obtained by measuring our internal lab standard (VOULEP) in the same analytical batch. The uncertainty thus obtained for δ^18^O (1σ) was 0.09‰ (n=4) and for δ D was 1.0‰.


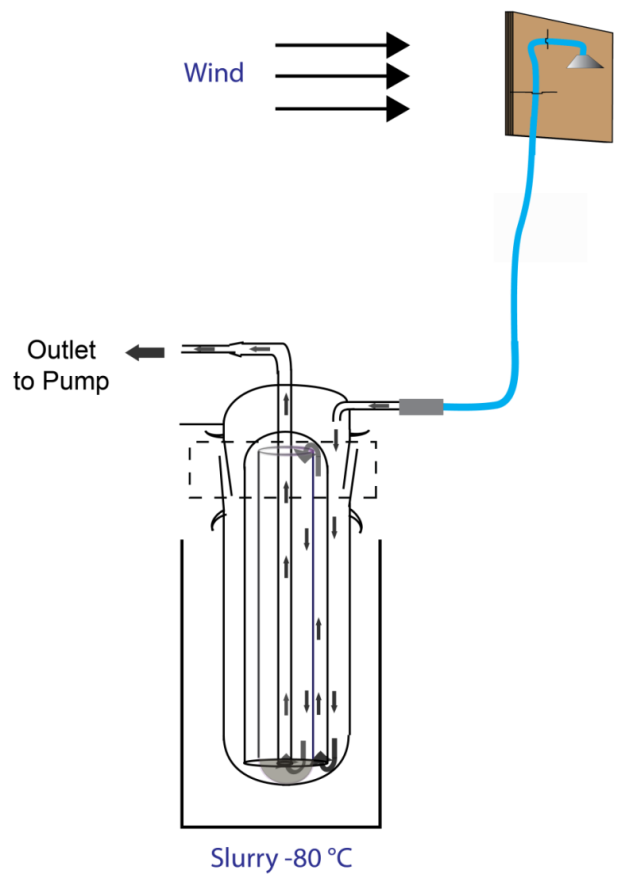


**Figure S2: Schematic of cold trap and its water vapor inlet connection used onboard Sagar Nidhi.**

**Table S1: Water vapor and rainwater samples collected during the Southern ocean expedition of 2013 for the period of January to February. The sample locations, date of sampling, and isotopic compositions are displayed in the table (δ^18^O, D and d-excess).**

**
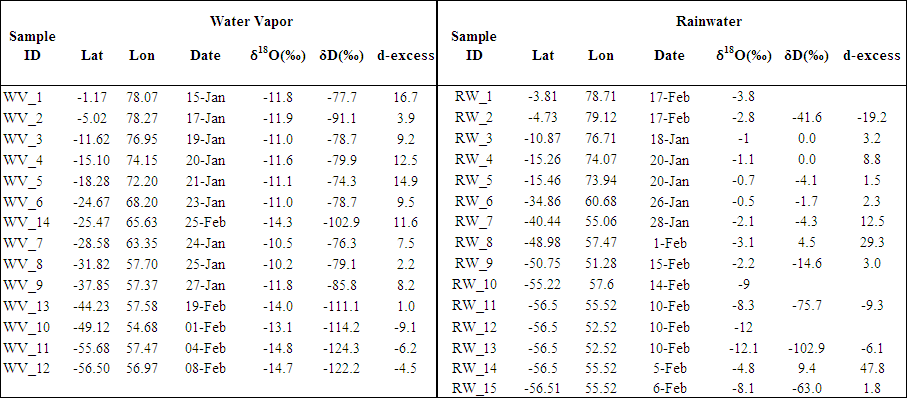
**

**2) Data used**

Single-Particle Lagrangian Integrated Trajectory (HYSPLIT) analysis from the National Oceanic and Atmospheric Administration (NOAA) Air Research Laboratory (<http://www.arl.noaa.gov/> ready/hysplit4.html) [*Rolph,* 2016; *Stein et al.,* 2015]. We obtained the air temperature data of multiple level and total precipitable water content (PWC) data over the Arabian Sea was obtained from National Center for Environmental Prediction/Department of Energy reanalysis (Reanalysis-2) data product of NOAA/OAR/ESRL PSD, Boulder, Colorado, USA, from their website at http://www.esrl.noaa.gov/psd/ [*Kanamitsu et al.,* 2002]. The SST data was obtained from National Oceanic and Atmospheric Administration (NOAA) optimum interpolation SST (OI SST) version 2 (V2) (<https://www.ncdc.noaa>. gov/oisst/data-access) with 1° × 1° spatial resolution [*Reynolds et al.,* 2002]. The rainfall (RF) data was obtained from Global Precipitation Climatology Project (GPCP) version 2.3 [*Huffman et al.,*2001].

**3) Trajectory analysis**

The Hybrid Single Particle Lagrangian Integrated Trajectory (HYSPLIT) model is used to understand the movement of air parcel using the lagrangian approach. The model also provides
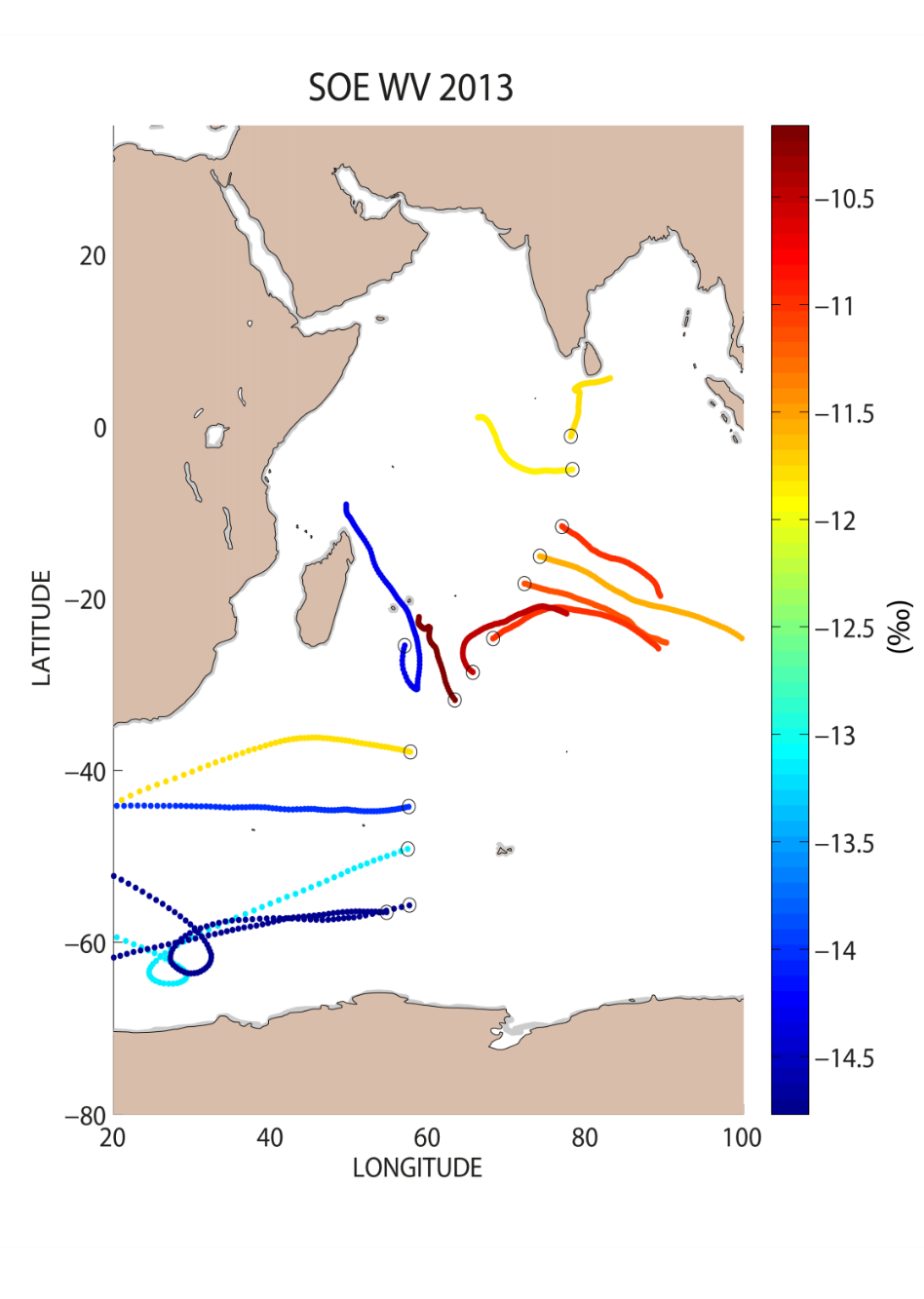


**Figure S3: The HySPLIT (Hybrid Single Particle Lagrangian Integrated Trajectory) 96 hours backward trajectories of air parcel reaching to the point of sampling are shown here. The black hollow circles indicate the sampling locations of water vapor. The color bar indicates 18O values in VSMOW that is used to represent the isotopic composition of samples for which the trajectories are obtained. *(The figure was generated using licensed*** [***MatlabR2014b (version 8.4)***](http://www.serc.iisc.in/facilities/matlab-8-4-r2014b/)***)***

meteorological variables as outputs along with the trajectory path: potential temperature, ambient temperature, precipitation, mixing depth, relative humidity, solar radiation, terrain height and altitude of the air parcel. The HYSPLIT trajectories corresponding to the days and locations of sampling are shown in **Figure S3** for a period of 96 hours**.** The directions agree in general with the prevailing wind direction at the time of sampling as displayed in **Figure 1c**. The trajectories are colored to represent the respective isotopic ratios measured in the water vapor samples collected at specific sampling station. The air trajectories north of 30°S indicate easterlies as dominant direction of trade winds whereas to the south of 30°S the westerlies were predominant.

**4) Comparison between modeled and observed rainwater isotopic composition**


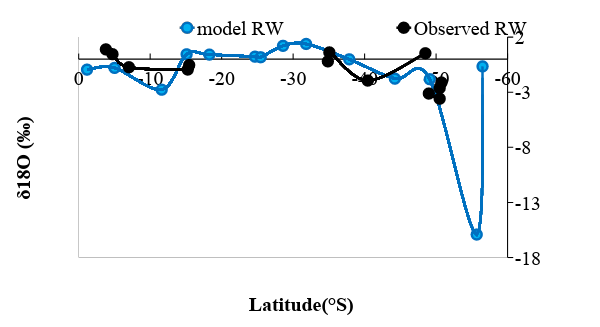


**Figure S4: The plot shows the comparison between modeled and observed rainwater (RW) isotopic composition across latitudes. The model calculations shows enriched sample over the Polar Regions while the most depleted samples are observed at 55S owing to high rainout fraction. The observation indicates good agreement with the calculations over the oceanic regions.**

**References**

Huffman, G.J., Adler, R.F., Morrissey, M.M., Bolvin, D.T., Curtis, S., Joyce, R., McGavock, B. and Susskind, J.,. Global precipitation at one-degree daily resolution from multisatellite observations. *Journal of Hydrometeorology*, *2*(1), pp.36-50(2001).

Rolph, G. D., Real-Time Environmental Applications and Display System (READY, NOAA Air Resour. Lab., College Park, Md. (2017) [Available at http://www.ready.noaa.gov.]

Stein, A. F., R. R. Draxler, G. D. Rolph, B. J. B. Stunder, M. D. Cohen, and F. Ngan, NOAA’S HYSPLIT atmospheric transport and dispersion modeling system, Bull. Am. Meteorol. Soc., 96(12), 2059–2077, doi:10.1175/bams-d-14-00110.1. (2015)

Kanamitsu, M., W. Ebisuzaki, J. Woollen, S. K. Yang, J. J. Hnilo, M. Fiorino, and G. L. Potter, NCEP-DOE AMIP-II reanalysis (R-2), Bull. Am.Meteorol. Soc., 83(11), 1631–1643, doi:10.1175/bams-83-11-1631. (2002)

Reynolds, R. W., N. A. Rayner, T. M. Smith, D. C. Stokes, and W. Q. Wang, An improved in situ and satellite SST analysis for climate,J. Clim., 15(13), 1609–1625, doi:10.1175/1520-0442(2002)015<1609:aiisas>2.0.co;2. (2002)

Rahul, P., P. Ghosh, S. K. Bhattacharya,and K. Yoshimura , Controlling factors of rainwater and water vapor isotopes at Bangalore, India: Constraints from observations in 2013 Indian monsoon, J. Geophys. Res. Atmos., 121, doi:10.1002/2016JD025352. (2016b).

Trenberth, K. E. Atmospheric moisture recycling: Role of advection and local evaporation. *Journal of Climate*, *12*(5), 1368-1381(1999).
